# The missing person in gene‐environment interactions

**DOI:** 10.1002/ggn2.10041

**Published:** 2021-03-04

**Authors:** Oluwatobiloba Osikoya, Myles Axton

**Affiliations:** ^1^ Advanced Genetics, Wiley Hoboken New Jersy USA

The personal environment is missing from our current methods in genetic epidemiology. Many individual journeys lead to the phenotypes of monogenic diseases so perhaps we should start to analyze the consequences of differences in the immediate environment for the diverse presentation of a single‐gene variant.

Genetics was supposed to move epidemiology beyond the original “Table 1 error” of assuming ancestry as surrogate genotype and ethnicity as surrogate environmental exposures—and to deliver precision in health prediction and healthcare.^1^ Without methods to prioritize research around a person's everyday exposures and experiences of disease at scale, even sophisticated genetic epidemiology will deliver only an outline of contributory factors. Indeed, genetic research might even worsen existing ancestry‐based health disparities in common and rare monogenic diseases.

Taking Sickle Cell Disease (SCD) as the classic example of a monogenic disease caused by a single mutation, a Perspective^2^ in this issue of the journal suggests how an international collaboration can take advantage of the range of individual experiences of SCD in resource rich—but unequal—and less well‐resourced environments to understand how a single mutation results in such a complex range of environmentally dependent experiences of disease and disability. At least for rarer but highly penetrant monogenic conditions with a small range of allelic variation that may be possible. Insights into the divergent phenotypes of SCD may be achieved by aggregating data globally to inform the research methods used to understand how gene‐environment interactions result in different health outcomes. These collaborative research efforts may in turn ameliorate the effects of health disparities for people with rare diseases or people in any population living with conditions where there is a strong genetic component. The proposed approach is rooted in the detailed experience of individuals sharing a genotype. To make it useful for research the reporting needs to be in units suitable for statistical methods. The duration or frequency of an episode of disease requiring intervention is a universal measure of disease experience that is useful across the diversity of reporting, adaptable to differential missingness in data, and useful despite the use of reporting instruments of types that greatly exceed the variation in genotyping technologies and analytic pipelines.

One of the insights in this Perspective is the conceptual use of individual health timelines recording the temporal correlation and intensity of factors influencing health status.^2^ These factors can be intrinsic (mutation, genotype, admixture) episodic (altitude, exercise, hospitalization) and continuing (geographic location, family, and community). There will be much redundancy and missingness in these datasets, but the aggregate will be rich in data to be mined for their patterns of correlation and causation. Since individuals with SCD will have most at stake in symptom mitigation, incentives for participation and guided self‐reporting should be introduced in the methodology in its design.

The authors have chosen pain to model which is apt because it is an important clinical and physiologic manifestation of SCD that is influenced by vast range of factors—not only environmental (eg, wind speed), behavioral (nutrition) and structural (governance) but sociocultural‐economic (family and social support).^2^ We expect from these heath timelines that subgroups of SCD disease course may emerge thereby pointing to a set of basic science models for mechanistic biology and therapeutic development toward personalized medicine.

At the genotype level, the Perspective's proposed approach complements, and may benefit from, statistical innovations that continue to extend the equity and utility of genome‐wide association studies (GWAS). For instance, by inferring the local genomic regions of different continental ancestry, admixed individuals have now been included in GWAS identifying and fine mapping variants conferring ancestry‐specific cardiovascular risk traits.^3^ Identifying genomic regions of local ancestry is relevant to research addressing gene‐environment interactions for diseases of both polygenic and single‐gene etiology. However, there is still a gap between ease in which genotypes can be interpreted and the multifaceted approaches to documenting exposures. In this respect, much more that could be made of the concept that nontransmitted alleles in relatives can be tested as genomic surrogates of the familial environment.^4^

The approach advocated in this Perspective is generalizable. Indeed, another monogenic disorder with variable clinical presentation, cystic fibrosis, has been analyzed for allele‐phenotype correlations for three traits: sweat chloride, lung function and pancreatic sufficiency.^5^ Still, relatively few of the environmental contributions to cystic fibrosis have been investigated (notably microbiota)—although genomic heterogeneity has been implicated in the inter‐individual variation in outcomes—for instance common loci influencing the lung function conferred by the commonest *CFTR* mutation.^6^ With the participation and insights of those people carrying the mutations, and with appropriate collaboration across geographic boundaries, it should be possible to capture the environmental influences that make the biggest differences to the experience of those affected by a shared genetic variant.

## AUTHOR CONTRIBUTIONS

**Oluwatobiloba Osikoya:** Writing‐original draft; writing‐review and editing. **Myles Axton:** Writing‐original draft; writing‐review and editing.

### PEER REVIEW

The peer review history for this article is available at https://publons.com/publon/10.1002/ggn2.10041.
